# Maternal representations in the dreams of pregnant women: a prospective comparative study

**DOI:** 10.3389/fpsyg.2013.00551

**Published:** 2013-08-27

**Authors:** Jessica Lara-Carrasco, Valérie Simard, Kadia Saint-Onge, Vickie Lamoureux-Tremblay, Tore Nielsen

**Affiliations:** ^1^Center for Advanced Research in Sleep Medicine, Hôpital du Sacré-Coeur de MontréalMontréal, QC, Canada; ^2^Department of Psychology, Université de MontréalMontréal, QC, Canada; ^3^Department of Psychology, Université de SherbrookeLongueuil, QC, Canada; ^4^Department of Psychiatry, Université de MontréalMontréal, QC, Canada

**Keywords:** dreaming, pregnancy, maternal mental representations, specificity of baby and child representations, pregnancy-related themes, dream emotions

## Abstract

Dreams are thought to respond to self- and socially-relevant situations that evoke strong emotions and require rapid adaptation. First pregnancy is such a situation during which maternal mental representations (MMR) of the unborn baby, the self and significant others undergo remodeling. Some studies suggest that dreams during pregnancy contain more MMR and are more dysphoric, but such studies contain important methodological flaws. We assessed whether dreamed MMR, like waking MMR, change from the 7th month of pregnancy to birth, and whether pregnancy–related themes and non-pregnancy characteristics are also transformed. Sixty non-pregnant and 59 pregnant women (37 early and 22 late 3rd trimester) completed demographic and psychological questionnaires and 14-day home dream logs. Dream reports were blindly rated according to four dream categories: (1) Dreamed MMR, (2) Quality of baby/child representations, (3) Pregnancy-related themes, (4) Non-pregnancy characteristics. Controlling for age, relationship and employment status, education level and state anxiety, women in both pregnant groups reported more dreams depicting themselves as a mother or with babies/children than did non-pregnant women (all *p* = 0.006). Baby/child representations were less specific in the late 3rd than in the early 3rd trimester (*p* = 0.005) and than in non-pregnant women (*p* = 0.01). Pregnant groups also had more pregnancy, childbirth and fetus themes (all *p* = 0.01). Childbirth content was higher in late than in early 3rd trimester (*p* = 0.01). Pregnant groups had more morbid elements than did the non-pregnant group (all *p* < 0.05). Dreaming during pregnancy appears to reflect daytime processes of remodeling MMR of the woman as a mother and of her unborn baby, and parallels a decline in the quality of baby/child representations in the last stage of pregnancy. More frequent morbid content in late pregnancy suggests that the psychological challenges of pregnancy are reflected in a generally more dysphoric emotional tone in dream content.

## Introduction

First pregnancy is an important transitional phase during which a mental reorganization leads to development of a woman's maternal identity and future maternal competencies (Ammaniti and Trentini, 2009; Slade et al., 2009). Through the activation of a caregiving system reciprocal to attachment (Solomon and George, 1996), this mental reorganization involves the elaboration and integration of maternal mental representations (MMR) of the unborn baby, of the woman as a mother, of non-maternal self-features, and of other significant relationships (Ammaniti and Trentini, 2009; Slade et al., 2009). However, while feelings of connection to and affection for the unborn baby increase throughout pregnancy, the woman's capacity to consider the fetus and herself as two autonomous and separate individuals is crucial to achieving a reciprocal and intimate relationship with the newborn child (Pines, 1972; Ammaniti and Trentini, 2009; Slade et al., 2009).

To date, empirical research indicates that the nature and qualities of MMR (e.g., their richness, specificity, and emotional tone) are principally rooted in the woman's internalized representations of the self and the other (Priel and Besser, 2001). They are also likely influenced by contextual factors such as actual relationships with her partner and family (Pajulo et al., 2001), psychological state (Pajulo et al., 2001; Theran et al., 2005), perceived fetal movements (Zeanah et al., 1990), ultrasound procedures (Viaux-Savelon et al., 2012), and high-risk environmental factors (Theran et al., 2005; Ammaniti et al., 2013). Time-dependent variations in the quality of MMR have also been described in several studies. Third trimester pregnant women have distinct, differentiated and emotionally invested MMR (Ammaniti et al., 1992), with a substantial presence of fearful imagery and worries about the child and the self as a mother (Vizziello et al., 1993; Leckman et al., 2004). These MMR display peak levels of richness and specificity by the 7th month of pregnancy with a subsequent decline up to childbirth (Stern, 1995; Innamorati et al., 2010). Stern (1995) interpreted this late decline as a need for the woman to “undo” her representations of the imagined child in order to prevent disappointment when finally face-to-face with the actual child.

Since the woman lacks information about her real baby, the development of MMR is thought to result from her projections, hopes, attributions, conscious and unconscious fantasies, and dreams (Stern, 1991, 1995; Ammaniti and Trentini, 2009; Slade et al., 2009). However, studies have principally investigated the more conscious aspects of MMR while giving surprisingly little attention to whether more unconscious processes, such as dreaming, might also contribute to their organization.

Dreaming is a form of mental activity occurring during sleep that can be recalled upon awakening. It is considered by some to be responsive to new emotion-evoking situations requiring adaptation, particularly situations involving close friends and family members (Cartwright, 2005, 2010; Nielsen and Lara-Carrasco, 2007). By linking memories and emotions in a less linear fashion than during waking thought (Hartmann, 1996), dreams are believed to connect recent emotional experiences to self-relevant memories and thereby to optimize psychological equilibrium and coherence of the self-system (Cartwright, 2005, 2010). In support of this possibility, content analyses have shown dreams to be highly social in nature, predominantly portraying interpersonal conflicts and concerns (Nielsen et al., 2004; McNamara and Szent-Imrey, 2007). Some clinical studies go further to support a regulatory function for dreaming in showing, for example, that clinically depressed divorcees who dream emotionally about their ex-spouses at intake are more likely to be psychologically well-adjusted several months later than are divorcees who do not report these types of dreams (Cartwright, 1991; Cartwright et al., 2006). Other studies with healthy subjects find that sadness dissipates across the night, a change that is positively associated with the number of intervening dream characters [for a review, see Kramer (2007)]. Based on such findings, it may be that life transitions involving the breaking or the (re)construction of significant relationships trigger representations of these relationships and associated emotions during dreaming, and that this socially structured dreaming facilitates the individual's adaptation to his/her new social context (Nielsen and Lara-Carrasco, 2007; Cartwright, 2010).

Accordingly, as pregnancy is an important period of mental reorganization about feelings, cognitions and relationships relating to the self and the unborn baby, pregnant women might well be expected to express these feelings, cognitions and relationships in their dreams. In this respect, a small number of systematic studies (Blake and Reimann, 1993; Van et al., 2004; Nielsen and Paquette, 2007) indicate that the vast majority of pregnant women (67–88%) report having at least one dream relating to a baby, pregnancy, or childbirth. Some others report that 30–62% of pregnant women's dreams refer to at least one of these maternal elements (Gillman, 1968; Van De Castle and Kinder, 1968; Winget and Kapp, 1972; Sered and Abramovitch, 1992) and that such dreams increase in frequency with advancing gestational age (Blake and Reimann, 1993). While such pregnancy dreams typically refer to the mother's physical well-being and to the sex of the unborn baby (Sered and Abramovitch, 1992), they also often contain elements of misfortune, injury or threat toward the baby, the mother or the father (Blake and Reimann, 1993; Van et al., 2004), and marital and familial issues (e.g., fear of losing the partner, dependency-independency issues with their own mother) (Van De Castle and Kinder, 1968). Other common themes relate to postpartum parental responsibilities and the fear of being an inadequate parent (Van De Castle and Kinder, 1968; Van et al., 2004). The few available comparative studies indicate that, relative to non-pregnant controls, pregnant women recall more dreams with pregnancy related themes (e.g., fetus, pregnancy, childbirth, one's own body, the baby's body) and more elements of danger toward the fetus and the self (Gillman, 1968; Van De Castle and Kinder, 1968; Dagan et al., 2001; Nielsen and Paquette, 2007). The dreams of pregnant women are also more negative (Gillman, 1968; Nielsen and Paquette, 2007) and contain more masochistic elements (i.e., misfortune, harm, environmental threats) but not more aggressive acts (Gillman, 1968).

In sum, though limited in number, studies provide ample evidence that MMR are expressed during dreaming. That MMR are frequently very emotional in nature provides some support for the suggestion that they are remodeled during dreaming and possibly serve a function in regulating emotions associated with this important developmental transition. Masochistic dreams have, in fact, been related to better outcomes, such as shorter labor durations (Mancuso et al., 2008) and less depressed mood 6–10 weeks postpartum (Kron and Brosh, 2003).

Nonetheless, most of the studies reviewed here contain important methodological flaws. First, prospective and longitudinal studies are notably lacking; existing studies are based largely on retrospective methods of dream collection that favor the recall of bizarre and intense dreams over more emotionally representative dreams (Schredl, 2010). Also, existing prospective studies exploit very small samples or fail to assess dream content related specifically to MMR; almost none control for potential confounders that might affect dream content or emotions, such as demographic characteristics and psychological state. To illustrate, masochistic dreams characterize depression-prone subjects and co-vary with the severity of depressed mood (Agargun, 2010); thus, as pregnancy is a period of increased risk for depressive disorders (Marcus, 2009), the finding of more negative dream elements during pregnancy needs to be replicated with appropriate controls for depressive mood. Finally, no available studies have examined whether dreamed MMR change in the last months of pregnancy as waking MMR do between 7 months and birth (Stern, 1995; Innamorati et al., 2010). Therefore, in addition to more prospective and carefully controlled studies, descriptive research is needed to clarify how dream content expresses MMR and how such content varies temporally toward the end of a first pregnancy.

### Objectives and hypotheses of the study

#### Objectives

This descriptive comparative study was part of a larger longitudinal study investigating the dream and sleep characteristics of 3rd trimester, nulliparous, pregnant women (i.e., at least 26 weeks or 7 months of gestation) and the capacity of these characteristics to predict delivery outcomes and postnatal depression.

In the present study, the first objective was to compare the prospectively collected dreams of nulliparous pregnant women, early and late 3rd trimester, with those of non-pregnant women on several measures designed to assess MMR in dreams. Several of these measures were based upon Stern's (1991, 1995) comprehensive theoretical conceptualization of mental organization specific to the mother-to-be that he terms the “motherhood constellation.” We also developed measures to assess the quality of representations of babies or children (i.e., the intensity of interaction with the mother and the level of separateness from the mother) and their valence (i.e., baby depicted negatively and/or as being in danger). Finally, we assessed whether the number of MMR and the quality of baby/child representations changed in the last stages of pregnancy (i.e., from the 7th to the 8th and 9th months of pregnancy).

The second objective of the study was to comparatively assess the frequencies of pregnancy-related dream themes (i.e., fetus, pregnancy, childbirth, human body) in order to replicate and correct methodological problems of previous studies. Whether these themes changed in frequency in the last stages of pregnancy was also explored.

The third objective was to examine non-MMR dream characteristics to determine whether more distal dream processes and characteristics are altered during pregnancy. These included dysphoric dream elements, dream interactions, and the capacity to report well-developed dream narratives (i.e., the dreamer's ability to form a dream connected to other memory material). Again, we explored whether these characteristics were subject to change throughout the last stages of pregnancy.

#### Hypotheses

1. We expected that the dreams of 3rd trimester pregnant women would contain more MMR, but not more representations of the self as a friend or in a work role. Because mental representations of the family have been found to gradually replace those of work and the pregnant woman's wider social context (Smith, 1999), representations related to friendship and work role were expected to be less frequent in their dreams. We also expected that dreamed MMR and representations of the self as friend or work colleague would be less prevalent among late than among early 3rd trimester pregnant women.
2. On the other hand, we expected that dream images about babies and children would be more specific and more negative among pregnant than among non-pregnant women, and among early than among late 3rd trimester pregnant women.
3. Finally, we expected dream characteristics relating to general emotions to differ between groups, with pregnant women having more dysphoric dream elements (e.g., morbid and masochistic elements), but not necessarily more negative dream interactions (e.g., lack of cooperation and aggressiveness). Also, since some clinical observations and self-report studies suggest that dream material is more accessible during pregnancy (Raphael-Leff, 1991; Lee and Dejoseph, 1992; Ablon, 1994; Kennedy et al., 2007), we expected that pregnant women would report better developed dream narratives.

## Materials and methods

### Participants

One hundred twenty-three healthy nulliparous women (62 3rd trimester pregnant women, 61 non-pregnant women) aged between 18 and 39 years were recruited by advertising, via health care centers of the province of Québec (Canada), and by word of mouth over a 4-month period (August to December 2010). They reported recalling at least one dream per week and being free from severe sleep and psychiatric disorders. None reported taking medications known to affect sleep. Pregnant women did not report any major obstetric complications. Participation in the study was on a voluntary basis requiring written consent with payment of $25 for expenses.

Two pregnant women and one non-pregnant woman were excluded because they failed to complete all questionnaires; one additional pregnant woman was excluded due to insufficient ability to understand and write French. The study sample thus included 59 pregnant and 60 non-pregnant women. Pregnant women were divided into two groups: early 3rd trimester (7 months or <30 weeks of gestation; *N* = 37) and late 3rd trimester (8–9 months or ≥30 weeks of gestation; *N* = 22) for dream content comparisons designed to examine the 7-month peak in MMR richness and specificity described earlier.

### Procedure

The research was conducted in the Dream and Nightmare Laboratory of the Hôpital du Sacré-Coeur de Montréal (Canada), an affiliate of the Université de Montréal, and was approved by scientific and ethical boards of the two institutions. After screening for inclusion and exclusion criteria on an initial phone interview, both groups of women received a set of questionnaires to complete at home and to return by mail to the laboratory when completed. The set included a custom demographics questionnaire, several psychological measures, and a prospective 14-day dream log (see details below).

### Demographic and psychological measures

#### Demographic questionnaire

All women completed a self-report questionnaire that included demographic information and in which they indicated any personal history of psychiatric problems on a single yes/no question (“I have had a major psychiatric disorder, e.g., mood disorders, anxiety disorders, schizophrenia, psychosis, etc.”).

#### State anxiety scale

Both groups completed the Spielberger State and Trait Anxiety Inventory (STAI) (Spielberger et al., 1970). The STAI is a widely used self-report measure of anxiety with good psychometric qualities in the general population (Spielberger, 1983) and among childbearing women (Grant et al., 2008). Only the state anxiety scale of the STAI was used in the present study. It consists of 20 items rated on 4-point scales evaluating emotional state at the time of the assessment (range 20–80). The scale had good internal consistency in the present study: Cronbach's α = 0.93 for pregnant women and.87 for non-pregnant women.

#### Depression scales

Depressive symptoms were assessed using the Edinburgh Postnatal Depression Scale (EPDS) (Cox et al., 1987) for pregnant women and the Beck Depression Inventory-Short Form (BDI-SF) (Beck et al., 1974) for non-pregnant women. Depression screening tools were different between groups since the EPDS is less reliant on somatic symptoms that are common during pregnancy (e.g., tiredness, appetite dysregulation) (Marcus, 2009). Both scales were used to classify women as having probable depressive disorders using cut-offs specific to each population. The EPDS is a widely used 10-item questionnaire assessing perinatal depression symptoms over the past 7 days using 4-point response scales (score range: 0–30). A score above 12 indicates probable depressive disorder with an overall sensitivity of 86% and specificity of 78% for all forms of (major and minor) depression (Cox et al., 1987). The questionnaire demonstrated acceptable internal consistency in the present study (Cronbach's α = 0.79). The BDI-SF is a 13-item questionnaire assessing depressive symptoms over the past 7 days using 4-point scales (score range: 0–39). A score above 8 screens moderate to severe depression in the general population with a sensitivity of 79% and a specificity of 77% (Nielsen and Williams, 1980). The scale had acceptable internal consistency in the present study (Cronbach's α = 0.77). Additionally, because dream emotions are known to be affected by pre-sleep mood (Schredl, 2010), both groups answered a 1–5 rating scale from the Sleep Disorders Questionnaire (Douglass et al., 1994) evaluating the occurrence of sadness/depressive feelings at bedtime (1 = never to 5 = all the time).

### Dream log

All women completed, for 14 consecutive days, a home dream log in which they were asked to describe up to three dreams per night (including naps). They were given a booklet containing 14 blank lined pages, each of which was followed by a second page containing questions assessing sleep and dream characteristics. Participants were instructed to write down their dreams immediately after awakening in the same order in which they occurred during the night. On the instruction page, they were informed that dreams were not always intense or easy to recall and that sometimes they could be simple sensory experiences, such as auditory, visual, or bodily impressions.

Overall, participants reported a total of 1795 dream narratives over the 14-day period (907 for pregnant women vs. 888 for non-pregnant women). Women indicated if there was no content to recall by checking the “no recall” box on the diary page. The number of dreams reported by a participant ranged from 4 to 29 for pregnant women (early 3rd trimester: *M* = 15.41, *SD* = 6.60; late 3rd trimester: *M* = 15.32, *SD* = 5.21) and from 3 to 27 for non-pregnant women (*M* = 14.80, *SD* = 5.63). Groups did not differ in the average number of dream reported per person (all *p* = ns). The mean number of words per dream was also calculated; a between groups analysis did not reveal any differences (non-pregnant: *M* = 94.92, *SD* = 5.44, early 3rd trimester: *M* = 95.96, *SD* = 6.93, late 3rd trimester: *M* = 81.80, *p* = ns).

### Dream content analysis

Dream reports were transcribed and presented in a randomized order to three raters (Jessica Lara-Carrasco, Vickie Lamoureux-Tremblay, and Kadia Saint-Onge) who were blind to the dreamer's pregnancy status and other information. To assess dream content specific to pregnancy, i.e., dreamed MMR, pregnancy-related themes and the quality of baby or child representations, dreams were scored using MMR and dream variables reviewed previously; other dream characteristics not specific to the pregnancy were assessed using validated scales for dream content analysis and one measure of psychological functioning applied to the dream narrative (see details in section Materials and Methods below). To assess reliability levels between raters, 40% of the dreams were scored by at least two raters. Interrater reliability (Cohen Kappa's coefficient, *k*) is reported for each variable described below.

#### Dream content specific to pregnancy

***Dreamed MMR***. Dreamed MMR were analyzed following the “motherhood constellation” model developed by Stern (1991, 1995), which was based on numerous theoretical writings and empirical findings in the fields of psychological process in motherhood and mother-infant attachment (e.g., Winnicott, 1965; Fraiberg et al., 1975; Bowlby, 1980). These “representations-of-being-with” include internalized sets of relationships concerning the baby (i.e., representation of the baby as a person, as having a type of personality or character), the woman (i.e., as a mother of this particular infant, as a wife to her husband, as a woman with a career, as a friend, as a daughter to her own parents, as a specific member of her original extended family), the baby's father, the woman's parents, other parental figures (e.g., grand-parents, uncles and aunts, cousins), family groupings (i.e., triads), and so on (Stern, 1991, 1995).

Dreams were rated for the presence (1) or absence (0) of each measure of the two following MMR categories: (1) *MMR characters:* representations of the dreamer's partner, mother, father, family (triads), and/or any baby or child (*k* = 0.63–0.95), and (2) *Social roles of the dreamer:* when the dreamer was represented as a character in her dream, her social attributes were scored (if applicable) as being any of the following: a mother, a spouse, a daughter, a member of her parents' family, a friend, and/or a worker/student (*k* = 0.47–0.85). Density scores were computed as follows: the number of each type of dreamed character was tallied and divided by the number of dreams reported across the 14 days, whereas the number each social role depicted was tallied and divided by the total number of occurrences of the dreamer across the 14 days. Extremely high *z*-scores were identified as univariate outliers on the following measures: occurrences of the dreamer's mother, father, and spouse and occurrences of the dreamer as a mother and as a worker/student; these were replaced by scores at the *z* = 3.29 limit.

***Quality of baby or child representations***. When a baby or a child image was present, its quality was evaluated on 1–9 *Likert* scales (1 = not at all to 9 = a lot) according to the *intensity of the dreamer*'*s interaction with the baby/child* (i.e., how much they interacted by gazing at, talking to, or touching each other) and to the *specificity and individuality of the personality of the baby/child* (i.e., how much the baby/child was described as having his/her own personality and as being an individual apart from the dreamer) (*k* = 0.60 and 0.52, respectively). Two other 1–9 *Likert* scales assessed the *affective valence of the baby/child representation* (1 = negatively depicted to 9 = positively depicted) and how much the *baby/child was depicted as being in danger* (1 = no danger is directed toward the baby at all to 9 = a lot of danger is directed toward the baby) (*k* = 0.59 and 0.66, respectively). Each of these four variables was averaged over the 14 journal days. There were no univariate outliers on these variables.

***Pregnancy-related themes***. The presence (1) or absence (0) of four pregnancy-related themes was evaluated: content relating specifically to pregnancy, childbirth, a fetus and/or the human body, regardless of whether the content made reference to the dreamer herself or to another dream character (*k* = 0.50–0.92). Each variable was tallied and divided by the number of dreams reported across the 14 days. Extremely high *z*-scores were identified as univariate outliers on the childbirth and fetus variables and were replaced by scores at the *z* = 3.29 limit.

#### Other dream characteristics not specific to pregnancy

Dream characteristics not specific to pregnancy included *development of the dream narrative*, which classifies each dream into one of five categories derived from the Dream-like Fantasy Scale (see Cartwright et al., 2003, 2006): 1 = no recall, 2 = a thought, 3 = a single image, 4 = a dream (two or more images with some connection between them), and 5 = a well-developed dream (more than two images with a well-developed plot) (*k* = 0.80). Higher scores (4 and 5) are defined to reflect the dreamer's ability to form a dream and connect it to other memory material (Cartwright et al., 2006). This scale co-varies with the severity of depressive mood and predicts positive psychological outcomes among depressed subjects (Cartwright et al., 2003, 2006). The scores for each dream were averaged over the 14 day-period.

Dream masochism was scored using the Masochism scale for dreams [see Winget and Kramer (1979) for an in-depth description of the scale] by assigning a binominal score to each dream as being either masochistic (1) or not (0) (*k* = 0.87; coefficient calculated on 10% of dream reports). A masochistic dream is defined as an unpleasant dream in which the dreamer has negative characteristics and/or the dream's outcome is negative. The dreamer is either depicted as less fortunate or less attractive than in reality (e.g., defective, ugly, sick), or is subjected to an unpleasant experience (e.g., thwarting, rejection, deprivation) (pp. 83–84 in Winget and Kramer, 1979). As mentioned earlier, dream masochism is correlated with current depressive state and vulnerability to depression (Agargun, 2010), but studies among childbearing women have also found masochistic dreams during pregnancy to predict better postnatal depressive mood (Kron and Brosh, 2003) and delivery outcomes (Mancuso et al., 2008). The number of masochistic dreams was divided by the total number of dreams reported over the 14 days.

Finally, *Aggressive (AG) and cooperative (COP) movements* and *morbid (MOR) dream content* were derived from the Special scores categories of the Exner scoring system for the Rorschach Inkblot test (Exner, 2003). The Exner scoring system has been applied to the scoring of dream narratives; human movements were found to successfully distinguish female and male adolescents' dream content (Winegar and Levin, 1997). Another study found AG, COP, and MOR categories to be reliable measures of psychological functioning when assessed in the dream content of children and adolescents living in situations of enduring violence (Kamphuis et al., 2008). For each dream report, the numbers of AG movements (dream action is clearly aggressive, including fighting, breaking, arguing, being angry, etc.), COP movements (interactions between two or more dream characters are clearly benevolent, cooperative or mutually supportive), and MOR elements (descriptions of dead, destroyed, damaged, polluted, degraded or broken dream elements, or a dysphoric feeling or character is attributed to a dream element) were tallied and divided by the number of dreams reported across the 14 days (*k* = 0.48–0.63).

### Data reduction and statistical analyses

To reduce the number of dependent measures and minimize multicollinearity between the dreamed MMR, a Principal Components Analysis (PCA) with varimax rotation, using an eigenvalue >1 extraction criterion with factor loadings >0.40, was conducted on the density scores for five MMR characters (any baby or child, her partner, her mother, her father, her family) that conceptually matched to the density scores of four social roles of the dreamer (dreamer as mother, spouse, daughter of her parents, member of her own family). The analysis yielded a four-factor solution explaining, altogether, 80.8% of the variance: (1) “as daughter-parents” (dreamer as daughter of her parents and dreamer's mother and father representations; 28.5% of explained variance), (2) “as spouse-partner” (dreamer as spouse and dreamer's partner representations; 25.9% of explained variance), (3) “as mother-baby/child” (dreamer as mother and representations of babies and children; 14.7% of explained variance), and (4) “as part of own family” (dreamer as part of her own family and representations of dreamer's family; 11.7% of explained variance). Higher scores on each of these four composite scores indicated more frequent dreamed MMR. Other social roles of the dreamer (as friend, as worker/student) were converted to *z*-scores and examined separately; these were not highly correlated with other dreamed MMR (absolute *r*'s ≤ 0.24).

Additionally, to avoid redundancy between the four variables relating to the quality of baby and child dreamed representations, a PCA with varimax rotation, using an eigenvalue >1 extraction criterion with factor loadings >0.40 was conducted, yielding a two-factor solution explaining altogether 70.1% of the variance: (1) “specificity of baby/child relationships and personality” (intensity of the dreamer's interaction with the baby/child, specificity and individuality of the personality of the baby/child; 38.9% of explained variance), and (2) “endangered and negative baby/child representations” (21.8% of explained variance). Higher scores on each of these two factors indicated more specific baby/child representations and greater endangered and negative baby/child representations, respectively. Table 1 summarizes and describes the final sets of dream variables.

**Table 1:** **Description of dream variables**.

**Dream variables**	**Description**
**1. DREAM CONTENT SPECIFIC TO PREGNANCY**	
**Dreamed maternal mental representations**	
As daughter-parents	Dreamer as daughter of her parents and dreamer's mother and father representations (factor score)
As spouse-partner	Dreamer as spouse and dreamer's partner representations (factor score)
As mother-baby/child	Dreamer as mother and representations of babies and children (factor score)
As part of own family	Dreamer as part of her own family and representations of dreamer's family (factor score)
As friend	Dreamer as friend (*z*-score)
As worker/student	Dreamer as worker or student (*z*-score)
**Quality of baby or child representations**	
Specificity of representations	Intensity of the dreamer's interaction with the baby/child, specificity and individuality of the personality of the baby/child (factor score)
Endangered and negative representations	Endangered and negative baby/child representations (factor score)
**Pregnancy-related themes**	
Pregnancy	Whether the content made reference to the dreamer herself or to another dream character (presence/absence; #occurrences/dream)
Childbirth	*Ibid*
Fetus	*Ibid*
Human body	*Ibid*
**2. DREAM CHARACTERISTICS NOT SPECIFIC TO PREGNANCY**	
Dream development^a^	1 = no recall, 2 = a thought, 3 = a single image, 4 = a dream (two or more images with some connection between them), 5 = a well-developed dream (more than two images with a well-developed plot)
Dream masochism^b^	An unpleasant dream in which the dreamer has negative characteristics and/or the dream's outcome is negative (yes/no; #occurrences/dream)
Aggressive movements^c^	Dream action is clearly aggressive, such as fighting, breaking, arguing, being angry, etc. (#movements/dream)
Cooperative movements^c^	Interactions between two or more dream characters are clearly benevolent, cooperative or mutually supportive (#movements/dream)
Morbid contents^c^	Descriptions of dead, destroyed, damaged, polluted, degraded or broken dream elements, or a dysphoric feeling or character is attributed to a dream element (#elements/dream)

a Categories derived from the Dream-like Fantasy Scale (see Cartwright et al., 2003 and Cartwright et al., 2006 for details).

b Masochism scale for dreams (see Winget and Kramer, 1979 for a detailed description).

c Categories derived from the Special scores categories of the Exner scoring system for the Rorschach Inkblot test (Exner, 2003).

In order to identify covariates to control in subsequent analyses, a series of one-way analyses of variance and chi-square tests compared groups of women (non-pregnant women, early 3rd trimester pregnant women, late 3rd trimester pregnant women) on demographic and psychological characteristics. Four sets of one-way multivariate analyses of covariance (MANCOVAs) with pregnancy status (non-pregnant women, early 3rd trimester pregnant women, late 3rd trimester pregnant women) as the between-group factor, and dream variables pertaining to each of the categories listed previously (i.e., “*Dreamed MMR,” “Quality of baby or child representations,” “Pregnancy-related themes,”* and “*Other dream characteristics”*) as multiple dependent variables, were performed controlling for demographic and psychological characteristics previously identified. Univariate effects were examined using Bonferroni adjustments (*p* = 0.05/number of comparisons) to control family-wise Type 1 errors (Tabachnick and Fidell, 2013). There were no multivariate outliers at α = 0.001 and assumptions of normality, linearity, and multicollinearity were all satisfactory. All analyses were performed with SPSS 20 (SPSS Inc., Chicago, IL, USA).

## Results

### Demographic and psychological characteristics

Non-pregnant women, early 3rd trimester and late 3rd trimester pregnant women differed significantly in age, relationship status, employment status, family income, education level and STAI state-anxiety score (see details in Table 2). However, family income did not remain significant when relationship status was controlled (non-pregnant: *M* = 4.30, *SE* = 0.33; early 3rd trimester: *M* = 5.28, *SE* = 0.40; late 3rd trimester: *M* = 5.59, *SE* = 0.50; *p* = 0.1). Thus, all these variables except family income were controlled in subsequent analyses.

**Table 2:** **Non-pregnant and pregnant (early and late 3rd trimester) women characteristics on demographic and psychological variables (mean ± standard deviation)**.

**Variables**	**Non-pregnant *N* = 60)**	**Early 3rd trimester *N* = 37)**	**Late 3rd trimester *N* = 22)**	***p*-value**
Age (years)	26.85 ± 4.17	27.84 ± 4.25	29.50 ± 4.01	0.04
Gestational age (weeks)		27.39 ± 1.14	33.48 ± 2.86	<0.001
**RELATIONSHIP STATUS**				
Single	35	0	0	<0.001
Married/in a relationship	25	37	22	
**EMPLOYED**				
Yes	42	24	17	0.6
No	18	13	5	
**EMPLOYMENT STATUS**				
Full-time	43	16	6	<0.001
Part-time	10	6	3	
Not	7	15	13	
working				
Family income^a^	3.80 ± 2.32	5.78 ± 2.51	6.09 ± 1.72	<0.001
Education^b^	7.95 ± 1.23	7.27 ± 1.81	7.23 ± 1.19	0.03
**MODERATE-TO-HIGH DEPRESSION^c^**				
Yes	3	4	1	0.5
No	57	33	21	
Depressed at bedtime^d^	1.62 ± 0.74	1.43 ± 0.56	1.64 ± 0.66	0.4
STAI-state anxiety	34.67 ± 7.62	31.79 ± 9.14	27.68 ± 7.94	0.003
**PERSONAL HISTORY OF PSYCHIATRIC PROBLEMS**				
Yes	5	7	2	0.3
No	55	30	20	

a Family income: 1 = $0–$10.000 to 8 = $75.000$ and up.

b Educational level: 1 = did not complete elementary school degree to 10 = completed a Ph.D. degree.

c Cut-off scores: EPDS > 12 and BDI-SF >8.

d Depressed at bed time: 1 = never to 5 = all the time.

### Non-pregnant and pregnant women (early and late 3rd trimester) differences on dream variables

Univariate analyses for dream content specific and not specific to pregnancy are detailed in Table 3.

**Table 3:** **Non-pregnant and pregnant women (early and late 3rd trimester) differences on dream variables (adjusted mean ± standard error)**.

**Dream variables**	**Non-pregnant (*N* = 60)**	**Early 3rd trimester (*N* = 37)**	**Late 3rd trimester (*N* = 22)**	***F***	***p*-value^†^**
**DREAM CONTENT SPECIFIC TO PREGNANCY**					
**Dreamed maternal mental representations^1^**					
As daughter-parents	−0.14 ± 0.16	0.20 ± 0.18	0.04 ± 0.25	0.81	0.4
As spouse-partner	0.02 ± 0.15	−0.18 ± 0.17	0.25 ± 0.23	1.46	0.2
As mother-baby/child	−0.53 ± 0.14	0.68 ± 0.16	0.30 ± 0.22	12.51	<0.001
As part of own family	0.11 ± 0.16	−0.19 ± 0.19	0.02 ± 0.25	0.72	0.5
As friend	0.11 ± 0.16	−0.19 ± 0.19	0.02 ± 0.25	0.72	0.5
As worker/student	0.11 ± 0.16	0.08 ± 0.18	−0.44 ± 0.24	2.01	0.1
**Quality of baby or child representations^2^**					
Specificity of representations^a^	0.24 ± 0.20	0.14 ± 0.20	−0.71 ± 0.26	4.77	0.01
Endangered and negative representations^a^	−0.11 ± 0.22	0.11 ± 0.21	0.05 ± 0.28	0.19	0.8
**Pregnancy-related themes^3^**					
Pregnancy	0.02 ± 0.02	0.18 ± 0.02	0.15 ± 0.02	19.33	<0.001
Childbirth	0.001 ± 0.01	0.06 ± 0.01	0.10 ± 0.01	12.62	<0.001
Fetus	0.001 ± 0.02	0.02 ± 0.01	0.02 ± 0.01	5.24	0.007
Human body	0.09 ± 0.02	0.15 ± 0.02	0.15 ± 0.03	2.37	0.1
**Other dream characteristics not specific to pregnancy**					
Dream development^4^	2.21 ± 0.08	2.38 ± 0.09	2.30 ± 0.13	0.75	0.5
Dream masochism^5^	0.12 ± 0.03	0.06 ± 0.03	0.10 ± 0.04	1.22	0.3
Aggressive movements^6^	0.29 ± 0.03	0.29 ± 0.04	0.17 ± 0.05	2.46	0.09
Cooperative movements^6^	0.22 ± 0.03	0.27 ± 0.03	0.26 ± 0.04	0.66	0.5
Morbid contents^7^	0.16 ± 0.03	0.27 ± 0.03	0.31 ± 0.05	3.11	0.05

Groups were compared using MANCOVA controlling for age, relationship status, employment status, education and state-anxiety score.

1 Higher density scores indicated more frequent dreamed representations.

2 Higher scores on each of these two factor scores indicated more specific baby/child representations and greater endangered and negative baby/child representations, respectively.

3 Higher scores on each of these variables indicated more frequent pregnancy-related themes.

4 1 = no recall to 5 = a well-developed dream.

5 Occurrences/dream.

6 Movements/dream.

7 Elements/dream.

a Non-pregnant N = 37, early 3rd trimester N = 29, late 3rd trimester N = 19.

† Error = adjusted p ≤ 0.008 for “Dreamed MMR”, p ≤ 0.025 for “Quality of baby or child representations”, p ≤ 0.013 for “Pregnancy-related themes” and p ≤ 0.01 for “Other dream characteristics not specific to pregnancy”.

#### Dream content specific to pregnancy

***Dreamed MMR***. A MANCOVA including the four dreamed MMR factors (*“as daughter-parents,” “as spouse-partner,” “as mother-baby/child,” “as part of own family”*) and the two other social roles z-scores (i.e., *“as friend,” “as worker/student”*) as dependent measures, the three groups of women (*non-pregnant, early 3rd trimester, late 3rd trimester*) as a between-group factor, and previously identified demographic and psychological measures as covariates was performed (Bonferroni adjustment for univariate effects, *p* = 0.008; 0.05/6). Since the assumption of homogeneity of variance-covariance was not met (Box's mean = 95.98, *p* < 0.001), the more robust Pillai's criterion was chosen for significance testing.

**Figure 1:**
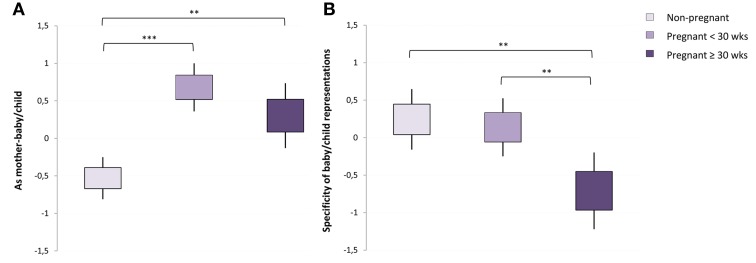
**Pregnant and non-pregnant women differences (mean ± standard error) on “As mother-baby/child” and “Specificity of baby/child representations” dream factor scores.** Early and late 3rd trimester pregnant women had more representations of themselves as mother and of babies and children **(A)** than did non-pregnant women in their dreams; late 3rd trimester pregnant women (≥30 weeks of gestation) had less specific babies and children representations in their dreams than did early 3rd trimester (<30 weeks of gestation) and non-pregnant women **(B)**. ^**^p < 0.01, ^***^p < 0.001.

Groups differed on the combined dependent measure [Pillai's trace = 0.27; *F*_(12, 214)_ = 2.73, *p* = 0.002, partial η^2^ = 0.13]. The only significant univariate effect was “*as mother-baby/child”* (*p* < 0.001, partial η^2^ = 0.18), which indicated higher scores for both groups of pregnant women than for non-pregnant women (all *p* = 0.006) (Figure 1A). There was also a trend for this score to be lower later in pregnancy; late 3rd trimester women had lower scores than did early 3rd trimester women (*p* = 0.1).

***Quality of baby/child representations***. As reported earlier, pregnant women differed from non-pregnant women in having significantly more babies and/or children depicted in their dreams. A total of 37 (61.7%) non-pregnant women, 29 (78.4%) early 3rd trimester women and 19 (86.4%) late 3rd trimester women, had dreams depicting babies and/or children for which the quality of the representations was scored.

A MANCOVA was conducted among this subgroup, with specificity of baby/child relationship and personality and endangered and negative baby/child representations as dependent measures, the three groups of women (*non-pregnant, early 3rd trimester, late 3rd trimester*) as a between-group factor, and previously identified demographic and psychological measures as covariates (Bonferroni adjustment for univariate effects at *p* = 0.025; 0.05/2). The assumption of homogeneity of variance-covariance was not met (Box's mean 14.86, *p* = 0.03) and the more robust Pillai's criterion was chosen for significance testing.

A marginal between group multivariate difference was found [Pillai's trace = 0.12; *F*_(4, 154)_= 2.35, *p* = 0.06, partial η^2^ = 0.05], with a univariate effect for the *specificity of baby/child relationship and personality* factor (*p* = 0.01, partial η^2^ = 0.11). As shown in Figure 1B, the late 3rd trimester group had less specific baby/child representations than did either the early 3rd trimester (*p* = 0.005) or the non-pregnant (*p* = 0.01) groups. The latter two groups did not differ (*p* = 0.7).

***Pregnancy-related themes***. A MANCOVA including the four pregnancy themes (pregnancy, childbirth, fetus, human body) as dependent measures, the three groups (*non-pregnant, early 3rd trimester, late 3rd trimester*) as the between-group factor, and previously identified demographic and psychological as covariates was performed (Bonferroni adjustment for univariate effects at *p* = 0.013; 0.05/4). The assumption of homogeneity of variance-covariance was satisfactory (Box's mean = 10.86, *p* = ns), so the more liberal Wilks λ criterion was chosen for significance testing.

**Figure 2:**
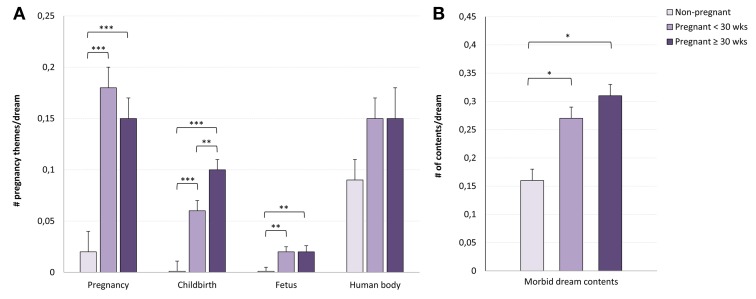
**Pregnant and non-pregnant women differences (mean ± standard error) on pregnancy-related themes and on morbid dream contents.** Early and late 3rd trimester pregnant women had more pregnancy, childbirth and fetus dream themes than did non-pregnant women, and late 3rd trimester (≥30 weeks of gestation) had more childbirth dream themes **(A)** than did early 3rd trimester (<30 weeks of gestation) women; morbid dream contents were more frequent in both pregnant groups than in the non-pregnant group **(B)**. ^*^p < 0.05, ^**^p < 0.01, ^***^p < 0.001.

Groups differed on the combined dependent measure [Wilks λ = 0.66; *F*_(8, 216)_= 6.18, *p* < 0.001, partial η^2^ = 0.19], and significant univariate effects were found for pregnancy (*p* < 0.001, partial η^2^ = 0.26), childbirth (*p* < 0.001, partial η^2^ = 0.19) and fetus (*p* = 0.007, partial η^2^ = 0.09), but not for human body (*p* = 0.1, partial η^2^ = 0.04). Pairwise comparisons indicated that both pregnant groups had more pregnancy related themes in their dreams than did non-pregnant women (all *p* ≤ 0.01) (Figure 2A). Pregnant groups also differed in the prevalence of childbirth content; the latter was higher in the late than in the early 3rd trimester group (*p* = 0.01). The two pregnancy groups did not differ on pregnancy and fetus themes (*p* = 0.3 and 0.8, respectively).

### Other dream characteristic not specific to pregnancy

A MANCOVA including the five dream characteristics not specific to pregnancy (*“dream development”, “AG movements”, “COP movements”, “MOR contents”*, *“dream masochism”*) as dependent measures, the three groups of women (*non-pregnant, early 3rd trimester, late 3rd trimester*) as a between-group factor, and previously identified demographic and psychological measures as covariates, was performed (Bonferroni adjustment for univariate effects *p* = 0.05/5 = 0.01). The assumption of homogeneity of variance-covariance was not met (Box's mean = 52.49, *p* = 0.02) and the more robust Pillai's criterion was chosen for significance testing.

Groups differed on the multivariate dependent measure [Pillai's trace = 0.16; *F*_(10, 216)_ = 1.93, *p* = 0.04, partial η^2^ = 0.09], but the only significant univariate effect was marginal and concerned morbid (MOR) dream content (*p* = 0.05, partial η^2^ = 0.05). As shown in Figure 2B, there were more morbid elements in dreams of the two pregnancy groups than in the dreams of non-pregnant women (*p* = 0.04 and 0.02 respectively). Post-hoc Pearson correlations between MOR dream content and STAI-state anxiety scores revealed that these two variables were not correlated for non-pregnant women (*r* = −0.17, *p* = 0.20), but were slightly correlated for early (*r* = 0.30, *p* = 0.07) and late 3rd trimester (*r* = 0.39, *p* = 0.07) pregnant women.

Further post-hoc analyses indicated that MOR dream content correlated positively with “as daughter-parents” (*r* = 0.27, *p* = 0.04) and “as part of family” (*r* = 0.25, *p* = 0.05) in non-pregnant women, with “specificity of baby/child representations” (*r* = 0.45, *p* = 0.01) and “endangered and negative baby/child representations” (*r* = 0.49, *p* = 0.007) in early 3rd trimester women, and with the “childbirth” theme (*r* = 0.67, *p* = 0.001) in late 3rd trimester women. MOR dream content was also correlated with “human body” themes in non-pregnant (*r* = 0.27, *p* = 0.04) and in early 3rd trimester (*r* = 0.38, *p* = 0.02) women.

## Discussion

The first objective of this study, to compare nulliparous non-pregnant women to early and late 3rd trimester pregnant women on dreamed MMR frequencies, resulted in relatively few differences. A composite factor that included representations of the woman as a mother and of babies and children was the only MMR factor to differentiate groups. As expected, 3rd trimester women scored higher on this factor than did non-pregnant women, thereby replicating earlier findings (Gillman, 1968; Van De Castle and Kinder, 1968; Winget and Kapp, 1972; Sered and Abramovitch, 1992; Blake and Reimann, 1993; Dagan et al., 2001; Van et al., 2004; Nielsen and Paquette, 2007). This result also generally supports the view that, in being focused principally on the maternal role during pregnancy, dreams are continuous with daytime concerns, thoughts, fantasies, and so forth (Schredl, 2012). They appear to mirror the ongoing daytime processes of remodeling MMR of the self as a mother and of the fetus as a future baby (Ammaniti and Trentini, 2009; Slade et al., 2009).

However, there was no evidence that pregnant women dreamed more about themselves in the role of a spouse, a daughter or a member of her own family, as might be expected from an early study reporting that dreams frequently depict marital and familial issues during pregnancy (Van De Castle and Kinder, 1968). There were also no differences in the frequencies of dreamer's representations of themselves in the roles of friend and of worker or student. The lack of differences in dreamed MMR relating to other aspects of the self and to relationships is surprising considering the vast clinical and empirical literature suggesting that these representations are also subject to remodeling during pregnancy. First, clinicians consider pregnancy as a major testing point for the mother-daughter relationship during which the woman begins to identify with her own mother while being confronted with the reactivation of unresolved mother-daughter conflicts (Pines, 1972; Raphael-Leff, 1991; Stern, 1998; Slade et al., 2009). The paternal representation is also thought to be reworked through a gradual renunciation of the “internal omniscient father” and the implantation of a relationship of adult equals between the woman and her father (Raphael-Leff, 1991). Second, a representational concept of triangular interactions of the woman with her partner and the couple's child is thought to develop even before the birth of the baby (Bürgin and Von Klitzing, 1995). Finally, the pregnant woman's representational world has been reported to gradually shift from a public world of work and wide social context to a more personal world of family (Smith, 1999). Our findings suggest that none of these changes is paralleled in dream content during pregnancy.

Rather, our results suggest that dream activity might be required in late pregnancy to process MMR relating exclusively to the mother-baby relationship. A small cohort study yielded results similar to ours in showing that 3rd trimester pregnant women dream more about babies, but not about the family or the partner, than do non-pregnant women (Dagan et al., 2001).

That we assessed dreams prospectively in a large cohort while simultaneously controlling many potentially confounding factors boosts our confidence in the accuracy and representativeness of these differences. Further, our statistical controls may account for the fact that we did not replicate some previous findings and clinical impressions. On the other hand, since it has been suggested that by the end of pregnancy a woman has generally reached reconciliation with her internalized relationships, particularly those relating to her own mother (Raphael-Leff, 1991; Trad, 1991), it may be that issues relating to mental representations of other aspects of herself and her relationships have been processed in dreams at earlier stages of pregnancy. This possibility is consistent with our finding that dreams depicting the woman as a daughter and as part of her own family along with parents and family representations were not associated with morbid elements among pregnant women whereas they were among non-pregnant women. Longitudinal studies are clearly needed to assess this possibility.

Another important finding of the present study is that among those woman who dreamed about babies or children, the quality of these representations was less specific in the late 3rd than in the early 3rd trimester and the non-pregnant women groups. These results concord with MMR studies showing that 3rd trimester women have specific and rich images about the unborn baby (Ammaniti et al., 1992) and that the quality of these representations declines up to childbirth (Stern, 1995; Innamorati et al., 2010). As Stern (1991, 1995) has suggested, this decline might reflect a need for the woman to “undo” her representations to prevent disappointment when faced with the “real child” after birth. Another explanation for the present finding is that, around the 30th week of gestation, the number of spontaneous fetal movements (Kurjak et al., 2005) and of nighttime micro-arousals evoked by fetal movements (Nishihara et al., 2008) decrease until birth. In line with the continuity hypothesis of dreaming, the lower quality of dreamed representations of babies and children in late 3rd trimester might parallel the decrease in daytime and nighttime perceived fetal movements.

Contrary to our expectations, however, specific representations of babies and children were not more negative during pregnancy, even though more specific and negative representations correlated with more morbid dream elements in early 3rd trimester pregnant women. This result partially supports findings that women's concerns during the last trimester are characterized by recurrent thoughts relating to the possibility that something bad may happen to the baby (Vizziello et al., 1993; Leckman et al., 2004) and with earlier studies showing that babies are commonly depicted as being in danger in pregnant women's dreams (Gillman, 1968; Van De Castle and Kinder, 1968; Blake and Reimann, 1993; Van et al., 2004; Nielsen and Paquette, 2007). The fact that such correlations were not found in late 3rd trimester might reflect the women's greater confidence in the issues of pregnancy and the baby's safety, since neonatal and maternal morbidity decrease sharply after 36 completed weeks of gestation (Escobar et al., 2006).

The second objective of this study, to comparatively measure the frequencies of pregnancy-related dream themes (i.e., fetus, pregnancy, childbirth, human body) and to assess whether these themes' frequencies were different among the two pregnancy groups, resulted in a number of noteworthy differences. Childbirth dream themes, which were more frequent in both pregnancy groups than in non-pregnant women, were also more prevalent among late than among early 3rd trimester women. In contrast, frequencies of pregnancy and fetus themes, and to a lesser extent human body themes remained high and stable among the two pregnant groups. The latter finding supports previous studies showing that pregnant women's dreams depict greater pregnancy and childbirth themes (Dagan et al., 2001; Nielsen and Paquette, 2007), but they add to this the notion that mental reorganization in the very last stage of pregnancy becomes more focused on preparations for delivery. This shift in focus might parallel the mother's reality of enduring more frequent medical appointments during the last weeks of pregnancy and of undergoing an upsurge of intense ambivalent feelings of excitement, fear and anxiety about the coming event (Raphael-Leff, 1991; Smith, 1999). In our study, dysphoric feelings toward the delivery in late pregnancy are reflected in the clear association between the childbirth dream theme and more morbid dream content exclusively in late 3rd trimester pregnant women.

The final objective of the study, to assess whether more general dream characteristics are altered during pregnancy, produced surprisingly few differences. Unexpectedly, the results showed that development of dream narratives and dream masochism did not differ between groups. However, that cooperative and aggressive interactions in dreams did not differ between groups parallels the findings of an earlier study (Gillman, 1968). In fact, the only measure not specific to pregnancy that differentiated the groups assessed morbid dream contents, that is, dysphoric feelings and negative characteristics attributed to any dream element. These were more prevalent and were marginally associated with more state anxiety among both pregnancy groups, even though our pregnant participants were considerably less anxious than our non-pregnant participants. Together, these results suggest that general dream processes not directly related to pregnancy remain relatively stable during pregnancy, but that the psychological challenges of pregnancy may be reflected indirectly in a more dysphoric emotional tone in dream content.

It is worth noting that our groups showed impressive high dream recall rates: pregnant and non-pregnant women reported on average one dream per day. Our dream log instructions might account for this effect: they stated that participants could report up to three dreams per night. This may have biased participants toward reporting an atypically high number of dreams. On the other hand, we also specified in the instructions that dreams are not always an intense or an easy to recall experience and that sometimes they may be simple sensory experiences such as auditory, visual, or somatic impressions. This definition, based on the inclusive definition of dreams proposed by Nielsen (2003), may also have led to an increased number of dream reports in our study.

### Implications for dream function during pregnancy

Altogether, the present results are consistent with the notion that mental reorganization during the 3rd trimester of a first pregnancy is focused principally on the future mother's construction of a new maternal identity and of representations of her unborn baby. This reorganization is likely to be achieved through activation of the caregiving system, a motivational mechanism that guides maternal behaviors and that derives from cognitive and affective representations shaped by the mother's own first relationship experiences (Solomon and George, 1996; Ammaniti and Trentini, 2009; Slade et al., 2009). This transitional process of change in self-concept during pregnancy might require the activation of specific representations of the mother-infant relationship during dreaming, possibly by virtue of dreaming facilitating the integration of recent and remote memories (Cartwright, 2005, 2010).

How dreaming achieves this may reside in its suggested capacity to integrate emotional experiences about life transitions and significant others into the memory system defining the self-concept. Indeed, as dream research has consistently found that dreams are sensitive to relational issues and changes, transitional periods implicating significant new relationships, such as pregnancy, might trigger the oneiric activation of these relationships and their associated emotions in an adaptive manner (Cartwright, 2005, 2010; Nielsen and Lara-Carrasco, 2007). Dreams may even function to selectively influence and promote attachment in unattached or insecurely attached adults by activating the mnemonic processes associated with development and revision of internal working models (Zborowski and McNamara, 1998; McNamara et al., 2001). We add to this the suggestion that pregnancy is a sufficient condition to activate the caregiving system during dreaming and thereby to consolidate maternal images about both the self as a mother and babies and children. In containing more morbid elements and in being more focused on the delivery process, particularly late in the 3rd trimester, dreams might thus be part of a “working through process” that enables pregnant women to be more psychologically prepared to face childbirth (Mancuso et al., 2008) and to adapt to the maternal role (Kron and Brosh, 2003). Whether the processing of these representations in dreams is predictive of real mother-infant interactions after birth, as was found in studies assessing prenatal waking thoughts, expectations and representations (Zeanah et al., 1994; Benoit et al., 1997; Siddiqui and Hagglof, 2000), needs to be assessed further in longitudinal studies.

However, as Blagrove (2011) has pointed out, a difficulty in investigating dream function is that current experimental designs are correlational rather than experimental. Accordingly, it has not been possible to experimentally manipulate dream incorporations, i.e., to randomly assign participants to incorporator vs. non-incorporator groups. We thus remain unable to demonstrate dream function in any causal sense. Future studies could assess whether the intentional recall of dreaming promotes insight and personal growth (Hobson and Schredl, 2011), an experimental effect that might be reflected during pregnancy in a better understanding of a mother's own parental concerns, relational changes, and modifications in self-concept.

### Conflict of interest statement

The authors declare that the research was conducted in the absence of any commercial or financial relationships that could be construed as a potential conflict of interest.

## References

[B1] Ablon S. L. (1994). The usefulness of dreams during pregnancy. Int. J. Psychoanal. 75(Pt 2), 291–299 8063485

[B2] Agargun M. Y. (2010). Rapid eye movement sleep interruption as a therapy for major depression, in Sleep and Mental Illness, eds Pandi-Perumal S. R. Kramer M. (New York, NY: Cambridge University Press), 222–224

[B3] Ammaniti M. Baumgartner E. Candelori C. Perucchini P. Pola M. Tambelli R. (1992). Representations and narratives during pregnancy. Infant Ment. Health J. 13, 167–182

[B4] Ammaniti M. Tambelli R. Odorisio F. (2013). Exploring maternal representations during pregnancy in normal and at-risk samples: the use of the interview of maternal representations during pregnancy. Infant Ment. Health J. 34, 1–10 10.1002/imhj.21357

[B5] Ammaniti M. Trentini C. (2009). How new knowledge about parenting reveals the neurobiological implications of intersubjectivity: a conceptual synthesis of recent research. Psychoanal. Dialogues 19, 537–555 10.1080/10481880903231951

[B6] Beck A. T. Rial W. Y. Rickels K. (1974). Short form of depression inventory: cross-validation. Psychol. Rep. 34, 1184–1186 4424377

[B72] Benoit D. Parker K. C. Zeanah C. H. (1997). Mothers' representations of their infants assessed prenatally: stability and association with infants' attachment classifications. J. Child Psychol. Psychiatry 38, 307–313 10.1111/j.1469-7610.1997.tb01515.x 9232477

[B7] Blagrove M. (2011). Distinguishing continuity/discontinuity, function, and insight when investigating dream content. Int. J. Dream Res. 4, 45–47

[B8] Blake R. L. Jr. Reimann J. (1993). The pregnancy-related dreams of pregnant women. J. Am. Board Fam. Pract. 6, 117–122 8452063

[B9] Bowlby J. (1980). Attachment and Loss. New York, NY: Basic Books

[B10] Bürgin D. Von Klitzing K. (1995). Prenatal representations and postnatal interactions of a threesome (mother, father, baby), in Psychosomatic Obstetrics and Gynaecology, eds Blitzer J. Stauber M. (Bologna: Monduzzi Editore), 185–191

[B11] Cartwright R. (2005). Dreaming as a mood-regulation system, in Principles and Practice of Sleep Medicine, 4th Edn., eds Kryger M. H. Roth T. William C. D. (Philadelphia, PA: Elsevier Saunders), 565–572

[B12] Cartwright R. Agargun M. Y. Kirkby J. Friedman J. K. (2006). Relation of dreams to waking concerns. Psychiatry Res. 141, 261–270 10.1016/j.psychres.2005.05.013 16497389

[B13] Cartwright R. Baehr E. Kirkby J. Pandi-Perumal S. R. Kabat J. (2003). REM sleep reduction, mood regulation and remission in untreated depression. Psychiatry Res. 121, 159–167 10.1016/S0165-1781(03)00236-1 14656450

[B14] Cartwright R. D. (1991). Dreams that work: the relation of dream incorporation to adaptation to stressful events. Dreaming 1, 3–9 10.1037/h0094312

[B15] Cartwright R. D. (2010). The Twenty-Four Hour Mind: The Role of Sleep and Dreaming in our Emotional Lives. New York, NY: Oxford University Press; US

[B16] Cox J. Holden J. Sagovsky R. (1987). Detection of postnatal depression: development of the 10-item Edinburgh postnatal depression scale. Br. J. Psychiatry 150, 782–786 10.1192/bjp.150.6.782 3651732

[B17] Dagan Y. Lapidot A. Eisenstein M. (2001). Women's dreams reported during first pregnancy. Psychiatry Clin. Neurosci. 55, 13–20 10.1046/j.1440-1819.2001.00778.x 11235851

[B18] Douglass A. B. Bornstein R. Nino-Murcia G. Keenan S. Miles L. Zarcone V. P. Jr. (1994). The sleep disorders questionnaire. I: Creation and multivariate structure of SDQ. Sleep 17, 160–167 8036370 10.1093/sleep/17.2.160

[B19] Escobar G. J. Clark R. H. Greene J. D. (2006). Short-term outcomes of infants born at 35 and 36 weeks gestation: we need to ask more questions. Semin. Perinatol. 30, 28–33 10.1053/j.semperi.2006.01.005 16549211

[B20] Exner J. E. Jr. (2003). The Rorschach: A Comprehensive System, 4th Edn. Hoboken, NJ: John Wiley and Sons Inc.

[B21] Fraiberg S. Adelson E. Shapiro V. (1975). Ghosts in the nursery. A psychoanalytic approach to the problems of impaired infant-mother relationships. J. Am. Acad. Child Psychiatry 14, 387–421 10.1016/S0002-7138(09)61442-4 1141566

[B22] Gillman R. D. (1968). The dreams of pregnant women and maternal adaptation. Am. J. Orthopsychiatry 38, 688–692 10.1111/j.1939-0025.1968.tb02438.x 5661552

[B23] Grant K. A. Mcmahon C. Austin M. P. (2008). Maternal anxiety during the transition to parenthood: a prospective study. J. Affect. Disord. 108, 101–111 10.1016/j.jad.2007.10.002 18001841

[B24] Hartmann E. (1996). Outline for a theory on the nature and functions of dreaming. Dreaming 6, 147–170 10.1037/h0094452

[B25] Hobson J. Schredl M. (2011). The continuity and discontinuity between waking and dreaming: a dialogue between Michael Schredl and Allan Hobson concerning the adequacy and completeness of these notions. Int. J. Dream Res. 4, 3–7

[B26] Innamorati M. Sarracino D. Dazzi N. (2010). Motherhood constellation and representational change in pregnancy. Infant Ment. Health J. 31, 379–396 10.1002/imhj.20261 28543078

[B27] Kamphuis J. H. Tuin N. Timmermans M. Punamaki R. L. (2008). Extending the *Rorschach trauma* content index and aggression indexes to dream narratives of children exposed to enduring violence: an exploratory study. J. Pers. Assess. 90, 578–584 10.1080/00223890802388558 18925499

[B28] Kennedy H. P. Gardiner A. Gay C. Lee K. A. (2007). Negotiating sleep: a qualitative study of new mothers. J. Perinat. Neonatal Nurs. 21, 114–122 10.1097/01.JPN.0000270628.51122.1d 17505231

[B29] Kramer M. (2007). The Dream Experience: A Systematic Exploration. New York, NY: Routledge/Taylor and Francis Group

[B30] Kron T. Brosh A. (2003). Can dreams during pregnancy predict postpartum depression. Dreaming 13, 67–81 10.1023/A:1023397908194

[B31] Kurjak A. Carrera J. Medic M. Azumendi G. Andonotopo W. Stanojevic M. (2005). The antenatal development of fetal behavioral patterns assessed by four-dimensional sonography. J. Matern. Fetal Neonatal Med. 17, 401–416 10.1080/14767050400029657 16009643

[B32] Leckman J. F. Feldman R. Swain J. E. Eicher V. Thompson N. Mayes L. C. (2004). Primary parental preoccupation: circuits, genes, and the crucial role of the environment. J. Neural Transm. 111, 753–771 10.1007/s00702-003-0067-x 15205997

[B33] Lee K. A. Dejoseph J. F. (1992). Sleep disturbances, vitality, and fatigue among a select group of employed childbearing women. Birth 19, 208–213 10.1111/j.1523-536X.1992.tb00404.x 1472269

[B34] Mancuso A. De Vivo A. Fanara G. Settineri S. Giacobbe A. Pizzo A. (2008). Emotional state and dreams in pregnant women. Psychiatry Res. 160, 380–386 10.1016/j.psychres.2007.06.005 18708267

[B35] Marcus S. M. (2009). Depression during pregnancy: rates, risks and consequences–Motherisk Update 2008. Can. J. Clin. Pharmacol. 16, e15–e22 19164843

[B36] McNamara P. Andresen J. Clark J. Zborowski M. Duffy C. A. (2001). Impact of attachment styles on dream recall and dream content: a test of the attachment hypothesis of REM sleep. J. Sleep Res. 10, 117–127 10.1046/j.1365-2869.2001.00244.x 11422726

[B37] McNamara P. Szent-Imrey R. (2007). Costly signaling theory of REM sleep and dreams. Evol. Psychol. 5, 28–44

[B38] Nielsen A. C. Williams T. A. (1980). Depression in ambulatory medical patients. Arch. Gen. Psychiatry 37, 999–1004 10.1001/archpsyc.1980.01780220037003 7416912

[B39] Nielsen T. A. (2003). A review of mentation in REM and NREM sleep:“Covert” REM sleep as a possible reconciliation of two opposing models, in Sleep and Dreaming: Scientific Advances and Reconsiderations, eds Pace-Schott E. F. Solms M. Blagrove M. Harnad S. (New York, NY: Cambridge University Press), 59–74

[B40] Nielsen T. A. Kuiken D. Alain G. Stenstrom P. Powell R. A. (2004). Immediate and delayed incorporations of events into dreams: further replication and implications for dream function. J. Sleep Res. 13, 327–336 10.1111/j.1365-2869.2004.00421.x 15560767

[B41] Nielsen T. Lara-Carrasco J. (2007). Nightmares, dreaming, and emotion regulation: a review, in The New Science of Dreaming: Vol. 2 Content, Recall, and Personality Correlates, eds Barrett D. McNamara P. (Westport, CT: Praeger Publishers/Greenwood Publishing Group), 253–284

[B42] Nielsen T. Paquette T. (2007). Dream-associated behaviors affecting pregnant and postpartum women. Sleep 30, 1162–1169 17910388 10.1093/sleep/30.9.1162 PMC1978400

[B43] Nishihara K. Horiuchi S. Eto H. Honda M. (2008). A long-term monitoring of fetal movement at home using a newly developed sensor: an introduction of maternal micro-arousals evoked by fetal movement during maternal sleep. Early Hum. Dev. 84, 595–603 10.1016/j.earlhumdev.2008.03.001 18450390

[B44] Pajulo M. Savonlahti E. Sourander A. Piha J. Helenius H. (2001). Prenatal maternal representations: mothers at psychosocial risk. Infant Ment. Health J. 22, 529–544 10.1002/imhj.1016

[B45] Pines D. (1972). Pregnancy and motherhood: interaction between fantasy and reality. Br. J. Med. Psychol. 45, 333–343 10.1111/j.2044-8341.1972.tb02216.x 4680578

[B46] Priel B. Besser A. (2001). Bridging the gap between attachment and object relations theories: a study of the transition to motherhood. Br. J. Med. Psychol. 74, 85–100 10.1348/000711201160821 11314905

[B47] Raphael-Leff J. (1991). Psychological Processes of Childbearing. London; New York: Chapman and Hall

[B48] Schredl M. (2010). Characteristics and contents of dreams. Int. Rev. Neurobiol. 92, 135–154 10.1016/S0074-7742(10)92007-2 20870066

[B49] Schredl M. (2012). Continuity in studying the continuity hypothesis of dreaming is needed. Int. J. Dream Res. 5, 1–8

[B50] Sered S. Abramovitch H. (1992). Pregnant dreaming: search for a typology of a proposed dream genre. Soc. Sci. Med. 34, 1405–1411 10.1016/0277-9536(92)90149-K 1529378

[B73] Siddiqui A. Hagglof B. (2000). Does maternal prenatal attachment predict postnatal mother-infant interaction? Early Hum. Dev. 59, 13–25 10.1016/S0378-3782(00)00076-1 10962164

[B51] Slade A. Cohen L. J. Sadler L. S. Miller M. (2009). The psychology and psychopathology of pregnancy: reorganization and transformation, in Handbook of Infant Mental Health, 3rd Edn., ed Zeanah C. H. (New York, NY: The Gilford Press), 22–39

[B52] Smith J. A. (1999). Identity development during the transition to motherhood: an interpretive phenomenological analysis. J. Reprod. Infant Psychol. 17, 281–299 10.1080/02646839908404595

[B53] Solomon J. George C. (1996). Defining the caregiving system: toward a theory of caregiving. Infant Ment. Health J. 17, 183–197

[B54] Spielberger C. D. (1983). Manual for the State-Trait Anxiety Inventory. Palo Alto, CA: Consulting Psychologists Press

[B55] Spielberger C. D. Gorsuch R. L. Luschene R. E. (1970). Test Manual for the State-Trait Anxiety Inventory (Self-Evaluation Questionnaire). Palo Alto, CA: Consulting Psychologists Press

[B56] Stern D. (1998). Mothers' emotional needs. Pediatrics 102, 1050–1052 9794964

[B57] Stern D. N. (1991). Maternal representations: a clinical and subjective phenomenological view. Infant Ment. Health J. 12, 174–186

[B58] Stern D. N. (1995). The Motherhood Constellation: A Unified View of Parent-Infant Psychotherapy. New York, NY: Basic Books; US

[B59] Tabachnick B. G. Fidell L. S. (2013). Using Multivariate Statistics. Boston, MA: Pearson Education

[B60] Theran S. A. Levendosky A. A. Bogat G. A. Huth-Bocks A. C. (2005). Stability and change in mothers' internal representations of their infants over time. Attach. Hum. Dev. 7, 253–268 10.1080/14616730500245609 16210238

[B61] Trad P. V. (1991). Adaptation to developmental transformations during the various phases of motherhood. J. Am. Acad. Psychoanal. 19, 403–421 1744019 10.1521/jaap.1.1991.19.3.403

[B62] Van De Castle R. Kinder P. (1968). Dream content during pregnancy. Psychophysiology 4, 375

[B63] Van P. Cage T. Shannon M. (2004). Big dreams, little sleep: dreams during pregnancy after prior pregnancy loss. Holist. Nurs. Pract. 18, 284–292 15624275 10.1097/00004650-200411000-00004

[B64] Viaux-Savelon S. Dommergues M. Rosenblum O. Bodeau N. Aidane E. Philippon O. (2012). Prenatal ultrasound screening: false positive soft markers may alter maternal representations and mother-infant interaction. PLoS ONE 7:e30935 10.1371/journal.pone.0030935 22292077 PMC3264650

[B65] Vizziello G. F. Antonioli M. E. Cocci V. Invernizzi R. (1993). From pregnancy to motherhood: the structure of representative and narrative change. Infant Ment. Health J. 14, 4–16

[B66] Winegar R. K. Levin R. (1997). Sex differences in the object representations in the dreams of adolescents. Sex Roles 36, 503–516 10.1007/BF02766687

[B67] Winget C. Kapp F. T. (1972). The relationship of the manifest content of dreams to duration of childbirth in primiparae. Psychosom. Med. 34, 313–320 5074957 10.1097/00006842-197207000-00005

[B68] Winget C. Kramer M. (1979). Dimensions of Dreams. Gainesville: University of Florida Press

[B69] Winnicott D. (1965). The Maturational Processes and the Facilitating Environment: Studies in the Theory of Emotional Development. Oxford, England: International Universities Press

[B70] Zborowski M. J. McNamara P. (1998). Attachment hypothesis of REM sleep: toward an integration of psychoanalysis, neuroscience, and evolutionary psychology and the implications for psychopathology research. Psychoanal. Psychol. 15, 115–140 10.1037/0736-9735.15.1.115

[B71] Zeanah C. H. Carr S. Wolk S. (1990). Fetal movements and the imagined baby of pregnancy: are they related. J. Rep. Infant Psychol. 8, 23–36 10.1080/02646839008403605

[B74] Zeanah C. H. Scheeringa M. Boris N. (1994). Parenting styles and risks in the vulnerable infant. Curr. Opin. Pediatr. 6, 406–410 10.1097/00008480-199408000-00009 7951660

